# Efficacy of medium-chain fatty acid salts distilled from coconut oil against two enteric pathogen challenges in weanling piglets

**DOI:** 10.1186/s40104-019-0393-y

**Published:** 2019-11-09

**Authors:** Paola López-Colom, Lorena Castillejos, Agustina Rodríguez-Sorrento, Mónica Puyalto, Juan José Mallo, Susana María Martín-Orúe

**Affiliations:** 1 Animal Nutrition and Welfare Service, Animal and Food Science Department, Facultat de Veterinària, Universitat Autònoma de Barcelona, Bellaterra, Spain; 2 Norel S.A, Madrid, Spain

**Keywords:** Enteric pathogen, Gut microbiota, Intestinal immunity, Medium-chain fatty acids, Weaning pig

## Abstract

**Background:**

The search for alternatives to antibiotics in pig production has increased the interest in natural resources with antimicrobial properties, such as medium-chain fatty acids (MCFA) as in-feed additives. This study evaluated the potential of a novel blend of MCFA salts (DIC) from distilled coconut oil with a lauric acid content to reduce enteropathogens and control intestinal diseases around weaning. Two experimental disease models were implemented in early-weaned piglets, consisting of two oral challenges: *Salmonella* Typhimurium (1.2 × 10^8^ CFU) or enterotoxigenic *Escherichia coli* (ETEC) F4 (1.5 × 10^9^ CFU). The parameters assessed were: animal performance, clinical signs, pathogen excretion, intestinal fermentation, immune-inflammatory response, and intestinal morphology.

**Results:**

The *Salmonella* challenge promoted an acute course of diarrhea, with most of the parameters responding to the challenge, whereas the ETEC F4 challenge promoted a mild clinical course. A consistent antipathogenic effect of DIC was observed in both trials in the hindgut, with reductions in *Salmonella* spp. plate counts in the cecum (*P* = 0.03) on d 8 post-inoculation (PI) (*Salmonella* trial), and of enterobacteria and total coliform counts in the ileum and colon (*P* < 0.10) on d 8 PI (ETEC F4 trial). When analyzing the entire colonic microbiota (16S rRNA gene sequencing), this additive tended (*P* = 0.13) to reduce the Firmicutes/Bacteroidetes ratio and enriched Fibrobacteres after the *Salmonella* challenge. In the ETEC F4 challenge, DIC prompted structural changes in the ecosystem with increases in *Dialister*, and a trend (*P* = 0.14) to increase the Veillonellaceae family. Other parameters such as the intestinal fermentation products or serum pro-inflammatory mediators were not modified by DIC supplementation, nor were the histological parameters. Only the intraepithelial lymphocyte (IEL) counts were lowered by DIC in animals challenged with *Salmonella* (*P* = 0.07). With ETEC F4, the IEL counts were higher with DIC on d 8 PI (*P* = 0.08).

**Conclusions:**

This study confirms the potential activity of this MCFA salts mixture to reduce intestinal colonization by opportunistic pathogens such as *Salmonella* or *E. coli* and its ability to modulate colonic microbiota. These changes could explain to some extent the local immune cell response at the ileal level.

## Background

In modern pig production, early weaning around 4 wk of life is associated with an abrupt dietary change and immature organ function, with a consequent reduction in growth. Thus, piglets around the post-weaning period are highly susceptible to enteric bacterial infections caused by opportunistic pathogens. The use of antibiotics and other medications to circumvent this critical period has been common in intensive systems; however, it has contributed to the development of antibiotic-resistant strains. Indeed, the last report from the European Food Safety Authority (EFSA) for the situation in 2015 documented an increase in multi-drug resistant (MDR) strains of *Escherichia coli* and *Salmonella* Typhimurium [1]. There is therefore an urgent need to search for alternatives that will diminish diarrhea outbreaks and help to reduce the use of therapeutic and prophylactic antibiotics.

Feeding strategies are one of the most commonly-used management factors for the improvement of gut health and function in newly weaned pigs. A wide range of bioactive ingredients have been proposed as tools to control enteric pathogens, to help reduce the incidence and severity of digestive problems associated with weaning [2]. Among them, medium-chain fatty acids (MCFA), considered fatty acids with a chain length of 6- to 12- carbon atoms, have been proposed as a potential alternative to antibiotics based on their long-known antibacterial activity [3]. In contrast to antimicrobial agents, MCFA have not shown evidence of acquiring resistance [4, 5]. Besides this, MCFA are an immediate source of energy due to their rapid passive absorption and digestion, which is of particular interest for the nutrition of young animals [6, 7]. They occur naturally as medium-chain triglycerides (MCT) in milk fat, as well as in other vegetable sources, such as coconut or palm oils and *Cuphea* seed oil [8, 9]. The most abundant MCFA in coconut is lauric acid (C12), representing up to 45% of the coconut fat content, followed by capric (C10) and caprylic (C8) acids [10]. C12 acid has been proven to have the greatest antimicrobial function among all MCFA [4, 11].

Several published works can be found substituting fat sources with purified MCFA or alternative natural sources rich in MCFA, leading to improvements in the performance of piglets around weaning [12, 13]. Studies with healthy pigs [14–16] have demonstrated positive effects on growth performance, digestibility, and intestinal microbiota. The antibacterial effect has also been widely demonstrated *in vitro* [3, 17], particularly against *Salmonella* or enterotoxigenic *E. coli* (ETEC), among other pathogens [18, 19]. However, to our knowledge, most of the *in vivo* studies testing the effect of MCFA against pathogens were performed in rodents [5, 20] and chickens [21]. Only two studies have tested the potential of MCFA in challenged pigs; one tested the control of *Salmonella* in fatteners under commercial conditions [22] and the other assessed the efficacy against an LPS challenge in an experimental trial with weanlings [23]. Within this context, the objective of this work was to evaluate the potential of a combination of sodium salts of medium-chain fatty acids distilled from coconut oil to enhance the gut health of weaned piglets in the face of diarrheic enteric diseases caused by *Salmonella* or ETEC F4 in controlled clinical assays.

## Methods

Two different experiments were performed to evaluate the effect of the coconut distillates against an oral challenge with *Salmonella* Typhimurium (Trial 1) or ETEC F4 (Trial 2). Both trials were performed at the Servei de Granges i Camps Experimentals of the Universitat Autònoma de Barcelona (UAB). The treatment, management, housing, husbandry and slaughtering conditions conformed to European Union Guidelines (Directive 2010/63/EU).

### Animals, housing, and experimental design

The two trials were conducted following Biosafety Level 2 requirements with appropriate training of the involved personnel. For the first trial, 56 male piglets from high sanitary status farms were used. The (Landrace × Large White) × Piétrain piglets originated from mothers with negative *Salmonella* serology results; they were weaned at 28 d of age and had an average body weight (BW) of 8.1 ± 1.16 kg. For the second trial, 72 male piglets (Landrace × Large White) × Piétrain that originated from mothers that were not vaccinated against *E. coli* were used; they were weaned at 21 d of age and had an average BW of 5.6 ± 0.95 kg.

The piglets were transported to the UAB facilities and for each trial, 48 animals were placed in 16 pens (three animals per pen). In each pen, the animals were distributed according to weight (a low-, an intermediate-, and a high-weighted animal) to obtain a final homogenous weight among pens. The experimental treatments were evenly distributed among the pens. The experimental treatments included a control treatment (CTR) consisting of a plain diet without additives and the same diet including a commercial blend of salts of MCFA distilled from coconut oil (Dicosan) at 3 kg/t (DIC). The rest of the animals were kept in a separate room and used as controls for the challenges (placebo groups, PLB). For the first trial, eight piglets were distributed among four pens (two animals per pen), and for the second trial 24 piglets were distributed among eight pens (three animals per pen). Both placebo groups received the same plain diet given to the CTR groups and were euthanized at d 4 post-inoculation (PI).

Each pen (3 m^2^) had a feeder and a water nipple to provide feed and water for *ad libitum* consumption. The weaning rooms were equipped with automatic heating and forced ventilation. The experiments were conducted during the autumn-winter seasons (September–October and February–March for Trial 1 and 2, respectively) with a mean room temperature of 30.8 ± 5.28 °C. Both trials were maintained under a 13 h light/11 h dark lighting regimen.

### Experimental products and diets

The evaluated feed-additive is commercially available (Dicosan) and was supplied by Norel S.A. (Madrid, Spain). It consisted of a mixture of sodium salts of fatty acid obtained from the distillates of coconut oil (67% crude fat and a fatty acid profile with 48.4% lauric acid, 18.6% myristic acid, 9.9% palmitic acid, 6.8% oleic acid, 6.2% caprylic acid, 5.8% capric acid, 3.6% stearic acid and 1.3% linoleic acid).

The basal diet (Table 1) was formulated to satisfy the nutrient requirement standards for pigs [24]. For each trial, the diet was manufactured in the same batch and the additive was mixed with the corresponding amount of feed to obtain the DIC diet. Dicosan was included according to the manufacturer’s recommended dosage (3 kg/t). The intended dose of the additive was not analytically confirmed in the diet, however, it was expected to provide 1.11 kg/t of lauric acid.

**Table 1:** Ingredient composition and chemical analysis of the basal diet on an as-fed basis

Ingredients, g/kg		
Maize	205	
Wheat	180	
Barley 2 row	170	
Extruded soybean	150	
Sweet whey-powder (cattle)	112	
Fishmeal LT	60.0	
Soybean meal 47	70.0	
Whey powder 50% fat	25.0	
Mono-calcium phosphate	6.5	
Calcium carbonate (CaCO_3_)	3.8	
*L*-Lysine HCl	4.5	
Vitamin-Mineral premix^a^	4.0	
*DL*-Methionine 99	2.6	
Sodium chloride (marine salt)	2.4	
*L*-Threonine	2.3	
*L*-Valine	1.5	
*L*-Tryptophan	0.7	
Calculated nutrients, g/kg		
Metabolizable energy, kcal/kg	3381	
Calcium	6.5	
Phosphorus	6.6	
Sodium	2.3	
Chloride	5.7	
Potassium	9.1	
Magnesium	1.4	
Analyzed nutrients, g/kg	*Salmonella* trial	ETEC F4 trial
Dry matter	902	915
Ash	54.2	48.2
Crude fat	59.4	66.5
Crude protein	198	195
Neutral detergent fiber	84.3	81.7
Acid detergent fiber	31.1	31.5

^a^Provided per kilogram of complete diet: 10,200 IU vitamin A, 2,100 IU vitamin D_3_, 39.9 mg vitamin E, 3 mg vitamin K_3_, 2 mg vitamin B_1_, 2.3 mg vitamin B_2_, 3 mg vitamin B_6_, 0.025 mg vitamin B_12_, 20 mg calcium pantothenate, 60 mg nicotinic acid, 0.1 mg biotin, 0.5 mg folic acid, 150 mg Fe, 156 mg Cu, 0.5 mg Co, 120 mg Zn, 49.8 mg Mn, 2 mg I, and 0.3 mg Se

### Bacterial strains

For the first trial, the *Salmonella* strain used for the oral challenge was a monophasic variant of *Salmonella* Typhimurium (4,5,12:i:-) with a ACSSuT-Ge resistance profile and phage type U302; it was isolated from a salmonellosis outbreak in fattening pigs in Spain (mainly enteric with sporadic septicemia) and provided by the Infectious Diseases Laboratory of UAB (ref. 301/99). The oral inoculum was prepared by overnight incubation at 37 °C and 250 r/min in buffered peptone water (BPW; Oxoid, Hampshire, UK) and diluted (1:20) with sterile phosphate buffered saline (PBS; Sigma-Aldrich, Madrid, Spain). The final inoculum concentration was 5.8 × 10^7^ CFU/mL. To confirm the doses, serial dilutions of the inoculum were cultured in tryptic soy agar (TSA; BD Difco, Heidelberg, Germany) by overnight incubation at 37 °C.

For the second trial, the enterotoxigenic *Escherichia coli* (ETEC) F4 strain used (positive for virulence factors F4ab, F4ac, LT, STb, and EAST1 and negative for F6, F18, F41, STa, VT1, VT2, and EAE) was isolated from 14-wk-old pigs and provided by the Diseases Laboratory of UAB (ref. 30/14-3). The oral inoculum was prepared by overnight incubation at 37 °C and 250 r/min in brain heart infusion (BHI; Oxoid). The final inoculum was 2.5 × 10^8^ CFU/mL. To confirm the doses, serial dilutions of the inoculum were cultured in Luria agar (LA; Laboratorios Conda, Torrejón de Ardoz, Spain) by overnight incubation at 37 °C.

In both trials, the placebo animals received the same amount of confirmed sterile broth inocula.

### Experimental procedure

The animals received the experimental diets *ad libitum* over 14 d in the *Salmonella* trial and 15 d in the ETEC F4 trial. After 6 and 7 d of adaptation in the first and second trials, respectively, the animals were orally challenged with the pathogen or the sterile broth. One pig per pen was euthanized on d 4 and 8 PI. The PLB animals were maintained until d 4 PI and thereafter euthanized following the same procedures performed on the challenged groups.

Fecal samples for microbiological analysis were aseptically collected from the heaviest piglet from each pen (*n* = 16) at arrival and after the adaptation period (d 0 PI), after spontaneous defecation or by rectal stimulation. After the adaptation period, the pathogenic bacteria inocula were administered by oral gavage to all animals as a single dose of *Salmonella* Typhimurium (1.2 × 10^8^ CFU) or ETEC F4 (1.48 × 10^9^ CFU) on d 7 and 8, respectively. In order to ensure that the stomach was full at the time of inoculation and to facilitate bacterial colonization, feed was withdrawn at 21:00 the previous day and provided once again 30 min before inoculation the following morning.

Individual BW and pen feed consumption were registered during the adaptation period. Body weight was further recorded on d 0, 4, and 8 PI in both trials, whereas feed consumption was recorded on d 0, 2, 4, and 8 PI for the *Salmonella* trial and d 0, 1, 2, 3, 4, 5, 7, and 8 PI for the ETEC F4 trial. The average daily gain (ADG) and average daily feed intake (ADFI) were calculated per pen, which was considered the experimental unit.

After the oral challenge, animals were checked daily for clinical signs to evaluate their status (i.e. dehydration, apathy, fecal score), always by the same individual. Mortality was also registered. No antibiotic treatment was administered to any of the animals in any of the experiments. The fecal score was assessed using a scale: 1 = solid and cloddy, 2 = soft with shape, 3 = very soft or viscous liquid, and 4 = watery or with blood. A fecal score was registered individually on d 0, 1, 2, 3, and 7 PI in the *Salmonella* trial and on d 0, 1, 2, 3, 5, and 7 PI in the ETEC F4 trial. Rectal temperature was assessed using a digital thermometer (Accu-Vet, Import Vet S.A., Centelles, Spain) on d 0 and 2 PI. Within the *Salmonella* trial, additional fecal samples for microbiological analysis were collected from the same animals on d 1, 3, and 7 PI.

On d 4 and 8 PI, one pig per pen was euthanized sequentially during the morning (between 08:00 and 14:00) for sampling. On d 4 PI, the animal from each pen with the mid-weight at the beginning of the experiment was selected, and on d 8 PI, the animal with the highest weight was selected. A 10-mL blood sample was obtained by venipuncture of the cranial vena cava using 10-mL tubes without anticoagulant (Aquisel, Madrid, Spain). Immediately, the animals received an intravenous injection of sodium pentobarbital (200 mg/kg BW; Euthasol, Esteve, Barcelona, Spain). Once dead, the animals were bled, the abdomen was immediately opened, and the intestinal tract was excised.

The digesta content from the ileum (5 cm from the ileal-cecal junction) and proximal colon (5 cm from the ceco-colic junction) was collected and homogenized, and the pH was immediately determined using a pH-meter calibrated on each day of use (Crison 52-32 electrode, Net Interlab, Madrid, Spain). Different aliquots were collected for different determinations. Samples of approximately 5 g were kept immediately on dry ice until being stored at -20°C for the further analysis of short-chain fatty acids (SCFA) and lactic acid, and similar aliquots were stored at - 80°C for the high-throughput sequencing (HTS) of colonic microbiota. Another set of samples were preserved in a H_2_SO_4_ solution (3 mL of digesta plus 3 mL of 0.2 mol/L H_2_SO_4_) and stored at -20°C until ammonia (NH_3_) determination.

In the ETEC F4 trial, additional aliquots (approximately 2 g) from the ileum and colon digesta were also kept on ice for microbiological analysis and were processed in less than 4 h; other similar aliquots were stored at -80°C for *E. coli* F4 quantification by real-time quantitative PCR (qPCR) and for the HTS of colonic microbiota. In parallel, to analyze the bacteria attached to the ileal mucus and epithelium, 15-cm long sections of the ileum were excised, washed thoroughly three times with sterile PBS, opened longitudinally, and scraped with a microscope glass slide to obtain mucosa samples. One aliquot was kept on ice for microbial analysis. In the case of the *Salmonella* trial, additional cecum digesta samples were collected, kept on ice, and processed in less than 4 h for *Salmonella* counts.

For the histological study, 3-cm long sections from the distal ileum were removed, opened longitudinally, washed thoroughly with sterile PBS, and fixed by immersion in a formaldehyde solution 3.7–4% (PanReac, Castellar del Vallès, Spain).

Blood samples were centrifuged (1500 × *g* for 15 min) and the obtained serum was divided into different aliquots and stored at - 20°C.

### Analytical procedures

Chemical analyses of the diets including the content of dry matter (DM), ash, crude protein, and diethyl ether extract were performed according to the Association of Official Agricultural Chemists standard procedures [25]. Neutral detergent fiber and acid detergent fiber were determined according to the method of Van Soest et al. [26].

For microbial counts of *Salmonella* spp. (Trial 1), the cecum content and feces were suspended in buffered peptone water (BPW; 1:10). The quantitative determination consisted of 10-fold serial dilutions in PBS seeded in Xylose-Lactose-Tergitol-4 agar (XLT4; Merck, Madrid, Spain) and a count of H_2_S positive colonies and morphology compatible with *Salmonella* spp. after 24 h incubation at 37 °C. With this scheme, animals were given a count level as follows: negative, for animals with no *Salmonella* growth at 10^2^ dilution (< 10^3^ CFU/g fresh matter [FM]); low, for animals with counts of 10^3^ to 10^4^ CFU/g FM; medium, for animals with counts of 10^5^ to 10^6^ CFU/g FM; and high, for animals with counts of 10^7^ to 10^8^ CFU/g FM.

For microbial counts of enterobacteria and total coliforms (Trial 2), the ileum and colon contents and feces were suspended in PBS (1:10), serially diluted in Lactated Ringer’s Solution (Sigma-Aldrich), and seeded in MacConkey agar and Chromogenic agar (Oxoid), respectively. The counts were read after 24 h of incubation at 37 °C.

In addition, for microbial molecular analysis, DNA was extracted from samples (approximately 250 mg) and purified using the commercial QIAamp DNA Stool Mini Kit (Qiagen, West Sussex, UK). Subsequently, its concentration and purity were checked using a NanoDrop 1000 Spectrophotometer (Thermo Fisher, Wilmington, DE). The protocol was followed, applying all recommended optimization steps, in order to improve bacterial cell rupture and purity. The DNA was finally eluted in 200 mL of Qiagen buffer AE and stored at - 80 °C until use.

*E. coli* F4 was quantified in colonic digesta and ileal scrapings by qPCR using SYBR green dye. qPCR targeting the gene coding the F4 fimbria of ETEC F4 was performed by modifying the procedure described by Gustavo Hermes et al. [27]; the changes consisted of using SYBR Green PCR Master Mix PCR (Applied Biosystems, Carlsbad, CA) and reducing the total volume of the reaction to 20 μL, which included 10 μL of 2× SYBR Green PCR Master Mix PCR buffer, 0.88 μL of each primer (12.5 μmol/L), and 5 μL of the DNA sample.

In the case of the HTS of the colonic microbiota in both trials, the V3-V4 region of 16S rRNA was targeted using MiSeq® Reagent Kit v2 (500 cycles; MiSeq from Illumina, San Diego, CA). The primers used in the construction of the libraries with amplicons of putative 460 bp were as follows:

- F-5′-TCGTCGGCAGCGTCAGATGTGTATAAGAGACAGCCTACGGGNGGCWGCAG
- R-5′-GTCTCGTGGGCTCGGAGATGTGTATAAGAGACAGGACTACHVGGGTATCTAATCC

The sequence reads of the 16S rRNA gene generated from the MiSeq Illumina system were processed using the QIIME v.1.9.1 pipeline [28] with default settings. The quality filter of the already demultiplexed sequences was performed at a maximum unacceptable Phred quality score of Q20. The resulting reads were clustered to operational taxonomic units (OTUs) using uclust with 97% sequence similarity and a subsampling pick open reference method [29] at 10%. Representative sequences were assigned to taxonomy against the bacterial 16S GreenGenes v.13.8 reference database [30] at a 90% confidence threshold, and sequence alignment and phylogenetic tree building were performed using uclust and FastTree, respectively. Thereafter, chimeric sequences were removed with ChimeraSlayer [31] using the default settings and further quality filtering consisted of removing singletons and OTUs with a relative abundance below 0.005% across all samples, as recommended by Bokulich et al. [32].

The ammonia (NH_3_) concentration was determined with the aid of a gas-sensitive electrode (Hach Co., Loveland, CO), combined with a digital voltmeter (Crison GLP 22, Crison Instruments S.A., Barcelona, Spain), modified from Barba-Vidal et al. [33]. Three grams of digesta preserved in H_2_SO_4_ (1:2) was centrifuged at 1372 × *g* for 10 min. A supernatant was obtained and neutralized with 1 mL of 10 mol/L NaOH to reach a pH of 11, while stirring and measuring the ammonia released as different voltages in mV.

The SCFA and lactic acid concentrations were determined based on the method described by Richardson et al. [34] and modified by Jensen et al. [35] using gas chromatography after submitting the samples to an acid-base treatment, followed by a diethyl ether extraction and derivatization with *N*-(tertbutyldimethylsilyl)-*N*-methyl-trifluoroacetamide (MBTSTFA) plus 1% *tert*-butyldimethylchlorosilane (TBDMCS) agent (Sigma-Aldrich).

Tissue samples for morphological measures were dehydrated and embedded in paraffin wax, sectioned to 4-μm thick slices and stained with hematoxylin and eosin. The morphological measurements (villus height and crypt depth, counts of intra-epithelial lymphocytes and goblet cells in the villus, and counts of mitoses in the crypt) of 10 different villus-crypt pairs were performed using a light microscope (Leica DM5000B, Jenoptik, Barcelona, Spain) fitted to CapturePro software (ProgRes®, Jenoptik, Barcelona, Spain), using the technique described by Nofrarías et al. [36].

Serum concentrations of tumor-necrosis factor-α (TNF-α) were determined using Quantikine Porcine TNF-α kits (R&D Systems, Minneapolis, MN); the pig-major acute-phase protein (Pig-MAP) concentration was determined using a sandwich-type ELISA (Pig MAP Kit ELISA, Pig CHAMP Pro Europe S.A., Segovia, Spain). In the *Salmonella* trial, serological antibodies of *Salmonella* were tested using ELISA Herdchek Swine *Salmonella* (IDEXX, Hoofdorp, Netherlands).

### Statistical analysis

The effect of the experimental treatments on the performance and slaughter measurements was determined using the free software R v.3.4.3, using the stats package [37] lm function for a one-way ANOVA, with diet as a fixed effect. For the microbial analysis, in Trial 2, enterobacteria and coliform counts were log_10_-transformed and submitted to the same one-way ANOVA analysis. In the case of Trial 1, *Salmonella* spp. count levels were analyzed with the stats package likelihood.test function for frequency analysis; the number of positive animals for *Salmonella* spp. followed the stats package glm function under a binomial distribution. The Pig-MAP concentration levels were also analyzed with the same frequency analysis, considering the normal range (0.3–1 mg/mL), borderline (1–2 mg/mL), and high levels (> 2 mg/mL) [38], and the number of animals with detectable copies of the pathogen F4 gene or quantifiable levels of fermentation products (SCFA and lactic acid) were analyzed under a binomial distribution.

The average daily feed intake, rectal temperature, and daily fecal consistency were assessed using the lme4 package [39] lmer function for an adjusted linear mixed model, with a treatment-by-time interaction term.

For all analyzed data, the pen was the experimental unit. The alpha level for the determination of significance for all the analyses was 0.05. Statistical trends were also considered for 0.05 < *P *< 0.10, unless otherwise indicated. Data are presented as means and residual standard error (RSE).

Biostatistics of quality-filtered sequences were performed using the open source software R v.3.4.3. Firstly, an OTU table was imported into R with the phyloseq package [40]; only OTUs that were shared among experimental groups were included and unique OTUs were excluded. Diversity and ordination (non-multidimensional scaling, NMDS) were analyzed at the OTU level using the vegan package [41]. The richness and alpha diversity were calculated with raw counts based on the Chao1 estimator [42] to estimate the number of taxa in the community, and the Shannon index [43], which considers the richness as well as the evenness. For beta diversity, the Whittaker distance matrix was calculated based on the relative abundances using the betadisper function. To compare any differential effects from treatments, an ANOVA was performed for richness and diversity. For ordination analysis, a dissimilarity matrix based on the Bray-Curtis distances was also calculated with relative abundances and two different fitting model analyses were applied. The function envfit, which fits centroids of class variable levels defined as a factor onto an ordination, was used, and the anosim function was used for the analysis of similarities (ANOSIM). Finally, a differential abundance analysis was performed with the OTU and taxa relative abundances under a zero-altered negative binomial or negative binomial model with pscl [44] and mass [45] packages, respectively, and corrected for the false-discovery rate (FDR).

## Results

In both studies, the animals showed a good health status on arrival, with most of the animals adapting to the facilities and feed positively. Both challenges promoted a clinical course of diarrhea in most of the animals, who were recovering spontaneously at the end of the study. Some casualties were registered in both trials. After the *Salmonella* challenge, one animal from the DIC group was found dead on d 4 PI and two animals from the DIC and CTR groups, respectively, were found dead on d 6 PI. Within the ETEC F4 challenge, one animal from the CTR group was found dead on d 1 PI and a second animal from the DIC group had to be euthanized following the clinical flowchart on d 3 PI. There were no statistical differences in the number of causalities between the treatments (*P* > 0.05) in any of the trials.

### Animal performance

The effects of the experimental treatments on animal performance are shown in Table 2. There were no significant effects on the final BW, ADFI, or ADG with the administration of DIC in any of the trials, except for a lower ADG (*P* = 0.032) for the DIC group than the CTR group during the second PI period (4–8 d PI) in the ETEC F4 trial. Regarding the performance response in the PLB groups, in the *Salmonella* trial the ADG was 33 ± 76.0 g/d during the adaptation week, and 306 ± 92.3 g/d during the 4-PI period, whereas in the ETEC F4, these values were 75 ± 42.9 g/d and 174 ± 63.8 g/d, respectively. The ADFIs observed in the first trial for the PLB group were 184 ± 26.4 g/d and 379 ± 71.2 g/d during the pre- and post-inoculation days, respectively, and those in the second trial for the PLB groups were 187 ± 59.8 g/d and 255 ± 24.6 g/d, respectively.

**Table 2:** Effect of the experimental diets on animal performance in the *Salmonella* and ETEC F4 trials

Items	*Salmonella* Trial				ETEC F4 Trial			
	CTR	DIC	RSE	*P*	CTR	DIC	RSE	*P*
BW, kg								
Initial	8.1	8.1	0.14	0.900	5.6	5.6	0.10	0.239
Final	10.8	9.9	0.12	0.164	7.8	7.6	0.69	0.553
ADFI, g/d								
PreI^a^	233	226	28.1	0.598	178	162	38.4	0.431
4 PI^b^	218	193	46.7	0.302	219	211	45.8	0.729
8 PI^c^	428	443	79.5	0.713	282	276	82.0	0.894
Overall^d^	285	278	41.5	0.766	221	211	46.6	0.664
ADG, g/d								
PreI^a^	122	109	60.7	0.675	77	78	41.6	0.986
4 PI^b^	-4	-38	108.7	0.541	129	117	90.5	0.796
8 PI^c^	326	316	162.6	0.903	260	164	80.6	0.032
Overall^d^	144	126	70.9	0.616	140	111	43.3	0.207

*ADFI* Average daily feed intake, *ADG* Average daily gain, *BW* Body weight, *PI* Post-inoculation day, RSE Residual standard error

^a^Pre-inoculation period from 0–7 d or 0–6 d before inoculation in the *Salmonella* and ETEC F4 trials, respectively

^b^Period from 0–4 d post-inoculation

^c^Period from 4–8 d post-inoculation

^d^Period from experimental day 1–14 or 1–15 in the *Salmonella* and ETEC F4 trials, respectively

### Clinical signs

**Fig. 1:**
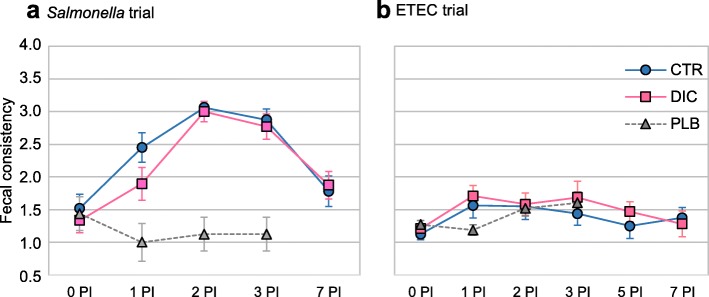
Fecal scores throughout the post-inoculation (PI) period in the *Salmonella* (**a**) and ETEC F4 (**b**) trials. The fecal score was measured using a scale from 0 (hard and cloddy) to 4 (watery or with blood). *CTR* control group, *DIC* Dicosan-supplemented group, *PI* post-inoculation day, *PLB* placebo group

The evolution of the fecal consistency throughout the post-inoculation (PI) period is presented in Fig. 1. After the oral challenge, fecal consistencies were impaired in both trials. The inoculation of *Salmonella* led to an evident liquid diarrhea (time *P* < 0.001), with scores reaching level 3 in most of the animals on d 2 PI. After the ETEC inoculation, diarrhea was observed (time *P* = 0.015) in a limited number of animals (barely reaching scores of 1.8). There were no significant differences related to the experimental diets for any of the trials (*P* > 0.31).

The *Salmonella* challenge increased the rectal temperature (40.6 ± 0.27 °C on d 2 PI vs. 39.4 ± 0.22 °C on d 0 PI; day *P* < 0.001). The ETEC F4 challenge did not affect this variable (39.1 ± 0.35 °C on d 2 PI vs. 39.0 ± 0.24 °C on d 0 PI; day *P* = 0.21). In relation to the effect of diets, DIC had no significant influence on rectal temperature (*P* > 0.6).

### Inflammatory response

After the *Salmonella* challenge, the CTR group presented higher concentrations of TNF-α (312 ± 129.2 pg/mL vs. 106 ± 15.9 pg/mL in PLB) and Pig-MAP (3.08 ± 2.812 mg/mL vs. 0.53 ± 0.140 mg/mL in PLB) on d 4 PI than the PLB groups. After the ETEC challenge, a higher response of both biomarkers was also observed in CTR groups than the PLB groups (156 ± 52.1 pg/mL in CTR vs. 123 ± 17.4 pg/mL in PLB, and 1.56 ± 1.545 mg/mL in CTR vs. 1.22 ± 1.055 mg/mL in PLB, for TNF-α and Pig-MAP, respectively). As expected, from day 4 to 8 PI, the TNF-α (149 ± 69.2 pg/mL and 113 ± 20.3 pg/mL for Trial 1 and 2, respectively) and Pig-MAP concentrations (0.96 ± 0.469 mg/mL and 1.46 ± 0.417 mg/mL for Trial 1 and 2, respectively) were decreased in the CTR groups. We were not able to detect statistical differences related to the experimental diets for any of the variables on any sampling day.

### Microbiological analysis

In the *Salmonella* trial, none of the animals seeded *Salmonella* in their feces on arrival nor after the 6 d of adaptation. The serological analysis from day 4 PI confirmed that the animals had not been exposed to *Salmonella* prior to the inoculation. PLB group remained negative for *Salmonella* spp. until d 4 PI. Only one piglet per group (CTR and DIC) seroconverted on d 8 PI by applying a cut-off of an optical density percentage (OD%) ≥ 40. Regarding the pathogen loads, all animals became positive, however, not all animals seeded countable numbers of the pathogen (> 10^3^ CFU/g FM). Supplementation with DIC reduced the number of animals with countable numbers in their cecum content, particularly on d 8 PI (Table 3).

**Table 3:** Effect of the experimental diets on the numbers of *Salmonella* spp.^a^ in the *Salmonella* trial

Items	*Salmonella* Trial		
	CTR	DIC	*P*
Feces			
1 PI	7/8	8/8	0.228
3 PI	8/8	6/8	0.080
7 PI	3/8	2/8	0.589
Cecum digesta			
4 PI	8/8	5/7	0.065
8 PI	7/8	3/8	0.033

*PI* post-inoculation day

^a^Number of animals with countable numbers of *Salmonella* spp. (< 10^3^ log_10_ CFU/g FM)

**Fig. 2:**
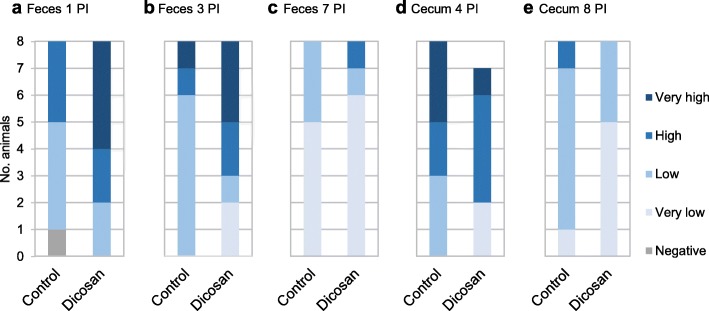
Number of animals with different levels of *Salmonella* spp. counts in feces and cecal contents. Feces sampled on d 1 (**a**), 3 (**b**), and 7 (**c**) post-inoculation (PI), and cecal content on d 4 (**d**) and 8 (**e**) PI (*Salmonella* trial). Range levels: negative (0 CFU/g), very low (< 10^3^ CFU/g), low (10^3^–10^4^ CFU/g), high (10^5^–10^6^ CFU/g), and very high (10^7^–10^8^ CFU/g)

Figure 2 shows the different levels of *Salmonella* spp. excretion in the feces and cecal content for the experimental diets. Although the differences were not statistically significant, there was a trend for DIC to increase the *Salmonella* counts in feces shortly after inoculation (d 1 PI, *P* = 0.10; and d 3 PI, *P* = 0.09), but to clear the pathogen more efficiently from the cecum on day 4 PI than the CTR group (*P* = 0.07). At the end of the study (d 8 PI), there was also an increase in the number of animals with unquantifiable counts (very low) in the cecum with the DIC treatment (63% vs. 12% for DIC and CTR, respectively; *P* = 0.26).

Regarding the ETEC F4 trial, there were no significant differences related to the experimental trials in the enterobacteria nor coliform plate counts in the feces upon arrival or from after the week of adaptation (9.77 ± 0.282 and 9.70 ± 0.360 log_10_ CFU/g FM, respectively). Table 4 shows the microbiological analysis on d 4 and 8 PI of the ileal and colonic digesta, and also of the ileal mucosa scrapings. On d 4 PI, the counts of both bacterial groups were not different between the diets. However, on d 8 PI, animals receiving DIC consistently had reduced numbers of enterobacteria and coliforms in their ileum and colon content compared to those who received the CTR treatment. This effect was particularly apparent in the ileum, with decreases of more than one log_10_ unit (5.38 vs. 6.71 log_10_ CFU/g FM, *P* = 0.004; and 5.33 vs. 6.51 log_10_ CFU/g FM, *P* = 0.05) for enterobacteria and coliforms, respectively. The attached enterobacteria in the ileal mucosa also tended to be reduced with DIC on d 8 PI compared to the CTR diets (*P* = 0.08).

**Table 4:** Effect of the experimental diets on enterobacteria and coliform counts in the ETEC F4 trial

Items	ETEC F4 trial			
	CTR	DIC	RSE	*P*
Ileum digesta				
Enterobacteria, log_10_ CFU/g FM				
4 PI	6.81	6.76	0.616	0.872
8 PI	6.71	5.38	0.783	0.004
Coliforms, log_10_ CFU/g FM				
4 PI	6.02	6.08	0.923	0.889
8 PI	6.51	5.33	0.829	0.013
Colon digesta				
Enterobacteria, log_10_ CFU/g FM				
4 PI	10.7	9.9	1.16	0.213
8 PI	10.9	9.9	1.03	0.083
Coliforms, log_10_ CFU/g FM				
4 PI	8.9	8.9	0.99	0.974
8 PI	10.8	9.8	0.98	0.052
Ileum mucosa				
Enterobacteria, log_10_ CFU/g FM				
4 PI	5.94	6.32	0.860	0.383
8 PI	6.17	5.16	0.786	0.082
Coliforms, log_10_ CFU/g FM				
4 PI	5.53	5.47	1.117	0.921
8 PI	5.48	5.09	1.093	0.489

*CFU* Colony-forming units, *FM* Fresh matter, *PI* Post-inoculation day, *RSE* Residual standard error

The pathogen was not always present at quantifiable levels (limit of detection 3.83 log_10_ F4 gene copies/g FM). A slight trend was found between the diets on d 4 PI in terms of the prevalence of the pathogen (*P* = 0.10), with all the CTR animals (8/8 animals) but not all from the DIC treatment (6/8 animals) being positive for *E. coli* F4 in the colon digesta. On day 8 PI, the pathogen presence was reduced in both groups, with no significant differences between them (*P* = 0.30; 2/8 vs. 4/8 animals for CTR and DIC, respectively), and similar loads (5.11 and 5.24 log_10_ F4 gene copies/g FM for the CTR and DIC groups, respectively). In the PLB group, the number of animals positive for *E. coli* F4 on day 4 PI was 5/8 with mean count of 3.23 log_10_ F4 gene copies/g FM, which is below the limit of detection.

### Microbiota 16S rRNA gene analysis

With the objective of assessing changes promoted by the additives to the intestinal microbiota, we analyzed colonic digesta samples taken on d 8 PI by high-throughput sequencing (HTS) of the V3-V4 regions of the 16S rRNA gene.

The microbiota analysis was performed using a more conservative approach, by using the OTU core, including only those species shared between experimental treatments, within each experiment (836/843 OTUs in Trial 1 and 833/848 OTUs in Trial 2). Regarding the evenness of the reads, similar values were obtained when comparing treated groups to CTR ones (*P* > 0.34; 4.94 ± 0.375 and 4.82 ± 0.243 log_10_ no. reads/sample in Trial 1 and Trial 2, respectively). The Chao1 index did not differ between the groups (*P* > 0.74).

**Fig. 3:**
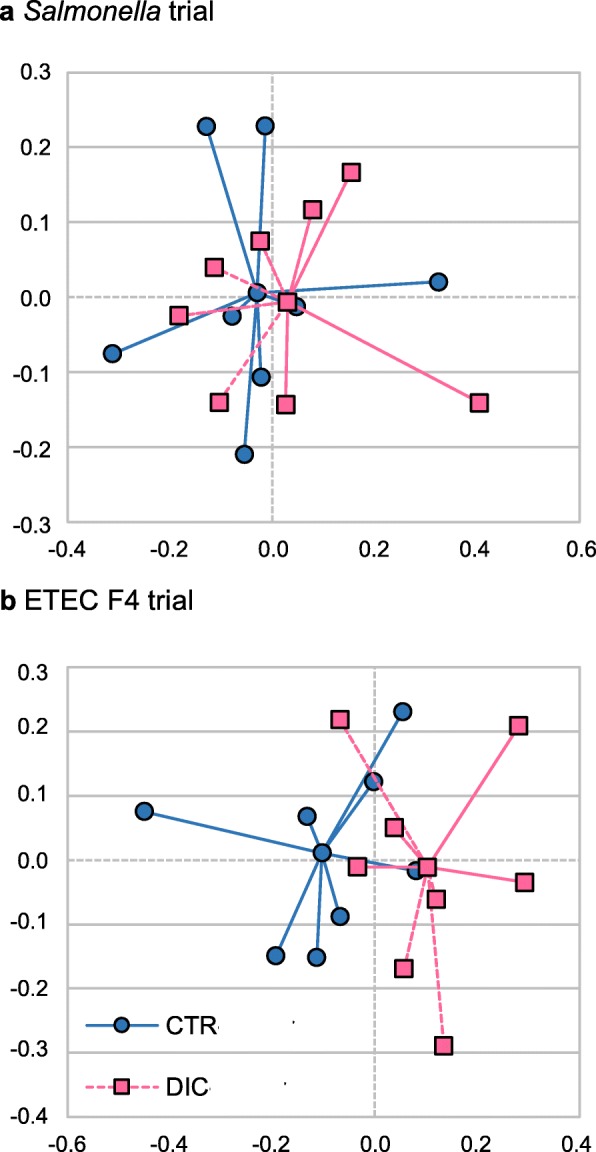
Non-metric dimensional scaling (NMDS) plot of the dissimilarity matrix based on Bray-Curtis distance. Clustering by experimental diets for the *Salmonella* (**a**) and ETEC trial (**b**) from colon digesta samples collected on day 8 post-inoculation (PI). *CTR* control group, *DIC* Dicosan-supplemented group

Regarding the microbial diversity or structure, DIC administration did not alter the alpha or beta diversity (Whittaker index) after *Salmonella* inoculation. After ETEC F4 inoculation, however, DIC administration did change the Bray–Curtis distances (microbial structure) as shown in Fig. 3 for the NMDS results (envfit *P* = 0.035 and ANOSIM *P* = 0.177).

Focusing on the aggregated taxa counts, Firmicutes and Bacteroidetes were the two major phyla (≥ 40%), with Proteobacteria following thereafter (7.3 ± 4.25% in the *Salmonella* trial and 9.1 ± 5.30% in the ETEC F4 trial). A few minor phyla (< 1%): Elusimicrobia, Fibrobacteres, Lentisphaerae, and Verrumicrobia, were encountered in the *Salmonella*-challenged animals.

The majority of the families observed in both trials were, by order of abundance: Prevotellaceae (27.9 ± 10.68% and 29.0 ± 7.52% in Trial 1 and 2, respectively), Veillonellaceae (22.7 ± 9.55 % and 15.4 ± 5.32 % in Trial 1 and 2, respectively), Rumminococcaceae (11.4 ± 3.42% and 11.4 ± 2.63% in Trial 1 and 2, respectively) and Lachnospiraceae (8.29 ± 3.555 % and 9.25 ± 3.307 % in Trial 1 and 2, respectively); 41 and 42 different families were detected per trial, respectively, representing approximately 5 % of the sum of the least abundant ones (< 1%, 31 families per trial). Among the genera detected, *Prevotella* was the most prevalent (28.0 ± 10.7% and 29.0 ± 7.52% in Trial 1 and 2, respectively). A total of 55 genera were found in the *Salmonella* and 54 in the ETEC F4 trials, and 40 and 42 genera were underrepresented (< 1%) in Trial 1 and 2, respectively, accounting for approximately 8 % of the total abundance. Unknown taxa represented 7.2 ± 2.87% and 6.7 ± 2.05 % of the families in the *Salmonella* and ETEC F4 trials, respectively, whereas they represented 24.8 ± 6.63 % and 27.2 ± 4.72% of the genera, respectively.

Regarding the influence of DIC, the Firmicutes:Bacteroidetes (F/B) ratio showed a trend of a numerical decrease (*P* = 0.132) in the *Salmonella* trial with DIC (1.14 vs. 1.47 in the CTR group). In the ETEC F4 trial, F/B ratio was unaffected (1.23 vs. 1.05 in the CTR group, *P* = 0.335).

The effects of DIC on specific populations are also presented in Table 5, considering only the taxa found in at least half of the samples. At the phylum level, under the *Salmonella* challenge, only the minor phylum Fibrobacteres (< 1%) was increased in DIC-supplemented animals compared to the CTR (*P* = 0.02) animals. In parallel, the corresponding family and genus, Fibrobacteraceae and *Fibrobacter*, respectively, were enriched in the DIC animals compared to the CTR animals (*P* < 0.05). Other changes in the *Salmonella* trial were also observed in minor families (< 1%), such as lower numbers of the candidate Barnesiellaceae and also in genera such as *Dialister*, *Lachnobacterium* and *Butyrivibrio* (*P* < 0.10). Although not significant, there was a relevant numerical decrease in the abundance of the Enterobacteriaceae family in DIC animals compared to in the CTR animals (*P* = 0.16).

**Table 5:** Ln changes in taxa promoted by DIC supplementation in the *Salmonella* and ETEC F4 trials

Taxonomic rank	Taxon	Ln change^a^ (DIC vs. CTR)	Adjusted *P*^b^
*Salmonella* trial			
Phylum	Fibrobacteres	2.229	0.019
Family	cand. Barnesiellaceae	-4.499	0.003
	Fibrobacteraceae	2.229	0.024
	Enterobacteriaceae	- 1.746	0.157
	Spirochaetaceae	1.326	0.192
	Alcaligenaceae	0.876	0.192
Genus	*Dialister*	- 0.690	0.000
	*Fibrobacter*	2.225	0.033
	*Lachnobacterium*	- 3.219	0.035
	*Butyrivibrio*	- 1.251	0.095
ETEC F4 trial			
Family	Veillonellaceae	0.363	0.136
	Oxalobacteraceae	- 0.804	0.151
	Deferribacteraceae	1.915	0.151
	Turicibacteraceae	- 1.384	0.176
Genus	*Dialister*	5.981	0.008
	*Lachnospira*	- 0.841	0.069
	*Megasphaera*	1.137	0.149
	*Coprococcus*	- 1.051	0.149
	*Oxalobacter*	- 0.804	0.173
	*Mucispirillum*	1.915	0.173
	*Turicibacter*	- 1.384	0.183

^a^Positive values and negative values indicate higher and lower abundance, respectively, in treated animals. Taxa are sorted by level of significance (from higher to lower). The presented differences are based only on taxa detected in at least half of the samples per diet

^b^Adjusted *P* value < 0.20

When considering the changes produced at the OTU level, i.e., species, only those found in at least half of the samples were considered. Table 6 presents the differences promoted by DIC (*P* < 0.05). As observed in the aggregated taxa, in the *Salmonella* trial, several OTUs assigned to Lachnospiraceae (new refs. OTU-2808 and 2343, and OTU-296082) were proportionally lower in DIC animals than CTR animals, including the *Lachnobacterium* genus (OTU-584463). In addition, a Spirochaetes-associated OTU was promoted in DIC animals (OTU-300859) and, in addition, several OTUs associated with *Prevotella* (within Bacteroidetes) were increased in DIC animals, possibly explaining the previously mentioned reduction in the F/B ratio. Nonetheless, other OTUs also corresponding to Bacteroidetes were in lower numbers, which highlights the high variability within a taxonomic group and the importance of considering the strain.

**Table 6:** Differentially abundant OTUs^a^ between the DIC and CTR groups in the *Salmonella* and ETEC F4 trials

Taxonomic classification^b^	OTU code	CTR	DIC
*Salmonella* trial			
Bacteroidetes; Prevotellaceae; *Prevotella*	300859	0.480	0.701
Firmicutes; Lachnospiraceae; *Lachnospira*	349257	0.273	0.175
Firmicutes; Clostridiales	New ref. 2497	0.005	0.089
Unassigned	New ref. 6586	0.005	0.062
Firmicutes; Erysipelotrichaceae	225636	0.055	0.002
Bacteroidetes; cand. Barnesiellaceae	315846	0.048	0.003
Bacteroidetes; Bacteroidaceae; *Bacteroides ovatus*	535375	0.039	0.015
Bacteroidetes; cand. Paraprevotellaceae; cand. *Prevotella*	New ref. 6366	0.065	0.052
Firmicutes; Christensenellaceae	410242	0.001	0.024
Spirochaetes; Sphaerochaetaceae; *Sphaerochaeta*	1832447	0.003	0.017
Bacteroidetes; Bacteroidales	New ref. 2311	0.003	0.025
Firmicutes; Clostridiales	357471	0.012	0.033
Bacteroidetes; Prevotellaceae; *Prevotella stercorea*	New ref. 1872	0.008	0.033
Bacteroidetes; Prevotellaceae; *Prevotella*	New ref. 1748	0.009	0.022
Bacteroidetes; Prevotellaceae; *Prevotella*	296082	0.008	0.023
Firmicutes; Lachnospiraceae; *Lachnobacterium*	584463	0.017	0.001
Bacteroidetes; Prevotellaceae; *Prevotella stercorea*	New ref. 2808	0.001	0.008
Bacteroidetes; cand. Paraprevotellaceae; cand. *Prevotella*	New ref. 648	0.002	0.009
Bacteroidetes; Prevotellaceae; *Prevotella stercorea*	302538	0.025	0.021
Firmicutes; Lachnospiraceae; *Blautia*	526773	0.014	0.013
ETEC trial			
Bacteroidetes; Prevotellaceae; *Prevotella stercorea*	524371	2.430	1.654
Firmicutes; Clostridiales	584083	0.210	0.261
Firmicutes; Veillonellaceae; *Megasphaera*	298050	0.038	0.220
Firmicutes; Lachnospiraceae; *Coprococcus*	344804	0.032	0.318
Firmicutes; Veillonellaceae; *Dialister*	403701	0.001	0.056
Bacteroidetes; Bacteroidaceae; *Bacteroides*	513445	0.006	0.084
Bacteroidetes; Prevotellaceae; *Prevotella*	248447	0.049	0.046
Bacteroidetes; S24-7	844589	0.004	0.040
Bacteroidetes; Bacteroidales; cand. Paraprevotellaceae; YRC22	289468	0.034	0.030
Bacteroidetes; Prevotellaceae; *Prevotella*	New ref. 2220	0.021	0.014
Firmicutes; Erysipelotrichaceae	287798	0.026	0.026
Firmicutes; Veillonellaceae; *Megasphaera*	264967	0.017	0.011
Bacteroidetes; cand. Paraprevotellaceae; cand. *Prevotella*	New ref. 5084	0.008	0.004
Firmicutes; Clostridiales	798164	0.010	0.011
Firmicutes; Ruminococcaceae	583134	0.008	0.013
Firmicutes; Lachnospiraceae; *Lachnospira*	843553	0.399	0.140
Firmicutes; Lachnospiraceae; *Coprococcus*	366623	0.282	0.000
Firmicutes; Clostridiales	826624	0.143	0.081

^a^Mean relative abundances (%) are presented for OTUs with an adjusted *P* value < 0.05 and detected in at least half of the animals per diet

^b^Order is specified only when it represents the lowest rank classification

When focusing on populations under the ETEC F4 challenge (Table 5), few changes were observed in DIC-supplemented animals; *Dialister* (*P* = 0.008) was significantly increased and *Lachnospira* tended to be reduced (*P* = 0.069) compared to CTR animals. Other bacteria were numerically altered (*P* < 0.20) by DIC, with most of them representing < 1%, with the exception of Veillonellaceae (*P* = 0.136; approximately 15%) and the genus within *Megasphaera* (*P* = 0.149; approximately 5%).

### Intestinal fermentation products

Table 7 shows the modifications of the pH and the main fermentation products in the ileum and colon digesta for both trials. The ileal pH was not significantly modified by the experimental treatments, except for a numerical reduction (7.00 vs. 7.20; *P* = 0.11) observed for DIC compared to CTR on d 4 PI in the *Salmonella* trial. In the ileum, the major product of fermentation was lactic acid, with ranges of 1.80–117 and 1.98–105 μmol/g FM in Trial 1 and 2, respectively. No differences were detected between the diets. Acetic acid was found at very low levels in the ileum in both trials (average values of 1.61 and 4.44 μmol/g FM for Trial 1 and 2, respectively), with several animals below the minimum detection level on d 8 PI (12 for Trial 1 and 3 for Trial 2), and no differences between the experimental diets.

**Table 7:** Effects of the experimental diets on intestinal fermentation parameters in the *Salmonella* and ETEC F4 trials

Items	*Salmonella* Trial				ETEC F4 Trial			
	CTR	DIC	RSE	*P*	CTR	DIC	RSE	*P*
Ileum digesta								
pH								
4 PI	7.20	7.00	0.228	0.111	6.81	6.80	0.208	0.891
8 PI	7.37	7.34	0.190	0.786	6.86	6.77	0.195	0.346
NH_3_, μmol/g								
4 PI	4.4	2.0	2.77	0.113	0.6	1.4	1.91	0.431
8 PI	14.6	19.1	8.89	0.326	1.0	1.1	0.39	0.752
Lactic acid, μmol/g								
4 PI	10.5	29.8	29.17	0.207	23.2	27.7	18.91	0.652
8 PI	41.8	23.4	27.09	0.196	19.1	36.0	25.73	0.209
Colon digesta								
pH								
4 PI	6.93	6.70	0.274	0.137	6.26	6.37	0.238	0.360
8 PI	7.07	7.04	0.156	0.671	5.97	6.07	0.266	0.464
NH_3_, μmol/g								
4 PI	32.5	24.4	19.94	0.431	10.7	10.8	5.821	0.969
8 PI	57.3	39.1	25.86	0.18	18.5	17.6	6.763	0.801
Total SCFA, μmol/g								
4 PI	98	88	37.1	0.613	107	102	21.5	0.606
8 PI	147	136	17.6	0.244	155	163	21.5	0.461
Acetic acid, %								
4 PI	55.8	55.2	8.22	0.892	60.5	60.1	6.01	0.907
8 PI	56.0	54.2	7.37	0.629	61.3	60.2	4.06	0.573
Propionic acid, %								
4 PI	26.6	24.6	3.86	0.317	22.5	24.7	5.91	0.479
8 PI	21.7	25.9	6.52	0.218	22.6	23.1	3.32	0.778
Butyric acid, %								
4 PI	12.9	14.3	6.01	0.649	12.6	11.2	3.78	0.492
8 PI	17.0	15.3	3.61	0.373	13.5	12.7	2.09	0.452
Valeric acid, %								
4 PI	2.65	4.10	1.804	0.118	2.73	2.55	1.327	0.802
8 PI	4.10	3.53	1.467	0.456	1.59	2.84	1.056	0.033
BCFA, %								
4 PI	2.16	1.83	1.186	0.586	1.68	1.37	0.774	0.465
8 PI	1.27	1.11	0.531	0.566	0.95	1.21	0.365	0.176

*BCFA* Branched-chain fatty acids, *PI* Post-inoculation day, *RSE* Residual standard error, *SCFA* Short-chain fatty acids

Regarding the colon digesta, there were no obvious changes in pH, despite a slight reduction with DIC on d 4 PI in the *Salmonella* trial (6.70 vs. 6.92; *P* = 0.14). The ammonia content was also not modified by the experimental diets. The total SCFA content was not modified by the experimental diets, but showed an increase from d 4 to 8 PI in both trials (from 92.7 to 141 and 104 to 159 μmol/g FM, respectively). The fermentation profile showed few changes with the diets; a numerical trend was observed in the *Salmonella* trial (*P* = 0.12) for an increase in the valeric acid proportion in the colon with DIC detected on d 4 PI. In the ETEC trial, higher valeric concentrations were also observed with DIC supplementation than with CTR diets on d 8 PI (*P* = 0.03).

### Ileal histomorphometry

Table 8 shows the changes promoted by the experimental diet in terms of ileal histomorphometry. The villus height showed a marked increase from d 4 to 8 PI in both trials, but was not modified by the experimental diets, neither were the crypt depth or the villus:crypt ratio. There were no differences related to the inclusion of DIC either for the number of mitoses or the number of goblet cells. However, intriguingly, the IEL counts displayed a trend to decrease with DIC in the *Salmonella* trial on d 8 PI (*P* = 0.07), whereas these were increased (*P* = 0.08) with the additive in the ETEC F4 trial.

**Table 8:** Effects of the experimental diets on histomorphometry in the ileum in the *Salmonella* and ETEC F4 trials

Items	*Salmonella* trial				ETEC F4 trial			
	CTR	DIC	RSE	*P*	CTR	DIC	RSE	*P*
Villus height, μm								
4 PI	185	183	65.5	0.962	278	229	89.4	0.289
8 PI	282	276	58.1	0.852	323	321	63.7	0.955
Crypt depth, μm								
4 PI	315	323	69.6	0.815	196	182	30.9	0.38
8 PI	300	290	37.1	0.596	189	184	19.7	0.600
Villus:Crypt								
4 PI	0.60	0.63	0.257	0.841	1.47	1.29	0.469	0.454
8 PI	0.99	0.99	0.209	0.944	1.78	1.79	0.364	0.935
MIT, cells/100 μm								
4 PI	0.251	0.325	0.160	0.39	0.078	0.068	0.103	0.845
8 PI	0.075	0.099	0.082	0.569	0.104	0.091	0.141	0.862
GC, cells/100 μm								
4 PI	2.20	1.93	0.851	0.556	1.75	1.43	0.542	0.262
8 PI	1.49	1.28	0.548	0.457	1.31	1.33	0.519	0.954
IEL, cells/100 μm								
4 PI	2.92	2.76	1.509	0.841	2.07	2.04	0.482	0.917
8 PI	3.92	3.06	0.882	0.072	2.39	2.88	0.523	0.080

*GC* Goblet cells in the villus, *IEL* Intraepithelial lymphocytes in the villus, *MIT* Mitoses in the crypt, *PI* Post-inoculation day, *RSE* Residual standard error

## Discussion

The present study aimed to evaluate the possible antimicrobial effect of a mixture of medium-chain fatty acids (MCFA) salts from coconut distillates (DIC) against enteric pathogens, such as *Salmonella* Typhimurium or ETEC F4, and the consequent reinforcement of the gut health and general health status of early-life weaned piglets. In order to fulfill these objectives, DIC was administered in feed in two different trials, each with one of the two pathogens. In both trials, animals exhibited signs of diarrhea after the oral challenge and an immediate decrease in feed intake.

During the week of adaptation after weaning, animals from the different diets displayed similar performances, with no influence of DIC supplementation. Other authors, however, have described improvements in performance related to the supplementation of MCFA. Some works evaluating MCFA as an energy source, at higher doses (2.1–7.75%), registered growth improvements [6, 13, 46, 47] that could be justified by the more rapid digestion and absorption of these fatty acids. Improvements have also been seen in animals challenged with ETEC F4 or LPS [23, 48], however, not necessarily by using high doses. Regarding the possible effects of MCFA supplementation on feed intake, we did not detect any impact of the experimental diets. The potential impact of MCFA as additives on feed intake is controversial. When supplemented at high levels, as a rapid source of energy, MCFA could prompt satiety due to their rapid oxidation in the liver [49, 50]. Moreover, MCFA have also been described as rancid and presenting low palatability [51, 52], which could therefore lead to a decline in feed intake and impair the adaptation to dry feed after weaning. Nonetheless, in our study, there was no negative impact of DIC on the feed intake during the first week post-weaning. The lower dosage (at 0.3%) than that used in other studies, and the different nature and composition of MCFA blends, could explain the discrepancies with other studies.

One of the most remarkable effects of this study was the specific action of supplemented MCFA on populations of *Salmonella*, coliforms, and enterobacteria. *Salmonella* spp. counts in the cecum tended to be reduced by DIC, despite a higher initial colonization after the challenge. After the ETEC F4 inoculation, DIC consistently diminished the enterobacteria and total coliform populations in the luminal content of the ileum and colon (particularly on d 8 PI), and the same trend was observed in the ileal mucosa, despite the effects on the luminal *E. coli* F4 pathogen being small. As stated in the introduction, a higher antibacterial activity has been described for MCFA than other free fatty acids [53]. Most recent studies have demonstrated their *in vitro* activity against a wide range of pathogenic bacteria [5, 11, 53, 54]. Lauric acid (C12) has been shown to have the highest antimicrobial activity among the fatty acids present in coconut oil [54, 55]. However, other medium-chain fatty acids, caprylic acid (C8) and capric acid (C10), showed the highest antimicrobial activity against different *E. coli* and *Salmonella* strains tested [3]. Freese et al. [56] and Sheu et al. [57] also reported lower minimum inhibitory concentrations (MIC) of caproic (C6) and caprylic (C8) acids for 50% growth inhibition of *E. coli* than capric acid (C10) and C12. Furthermore, C6 and C8 have been selected for their antimicrobial activity, rather than longer-chain fatty acids, to be tested in *in vivo* trials [23]. Nevertheless, although C12 is the major compound in the distillates of coconut (48.4% crude fat, CF), these also include important proportions of other MCFA (6.2% CF for C8 and 5.8% CF for C10) that might support the aforementioned effects.

Among the *in vivo* studies that have evaluated the effect of MCFA administration on pathogens, some authors have described lower numbers of clostridia or *E. coli* [15, 16, 58–60], and also *Salmonella* [22]. These reported effects on the colonization of the gut by different pathogens could have been mediated by the direct activity of MCFA, but also indirectly, by changes promoted by these additives in the intestinal microbiota. In this regard, different *in vivo* trials in pigs have described significant changes in bacterial populations promoted by the direct supplementation of MCFA supplementation [61, 62], including a transgenerational influence observed by the supplementation of sows having an effect on their offspring [63]. The variability in these effects in the literature could be the result of differences between MCFA sources, blends, dosages, and methods of administration, making the direct comparison between studies difficult. In particular, most of the experimental blends tested were composed of a combination of C8 and C10 and negligible quantities of C12, or their conjugated forms, into triacylglycerol (MCT), with lipases needed to release, not always successfully, the free fatty acids.

To our knowledge, very few works have evaluated the effect of MCFA on intestinal microbiota by performing an entire profiling of their populations, particularly when faced with a pathogen challenge. The results of our work show that, in global terms, the microbiota profiles observed after the two challenges were not substantially different from those previously described for piglets of this age [64, 65], suggesting that these animals did not suffer any drastic dysbiosis. In fact, other challenges in piglets with *Salmonella* could not differentiate clusters from unchallenged and *Salmonella*-challenged animals either [66], and were not even able to detect the *Salmonella* genus by HTS, as occurred in our animals. Nevertheless, it must be taken into account that the activity of the supplemented MCFA against potential pathogens was observed mainly in the ileum and cecum, and the assortment of microbial populations was also affected. This leads us to hypothesize that the direct impact on upper parts of the gut is reflected, for instance, on the gastric barrier or the small intestine in the foregut.

Nevertheless, there were still observed microbiota changes promoted by DIC diets. In both trials, the DIC diets were able to significantly modify particular microbial taxa, although the affected bacterial groups were different. With the *Salmonella* challenge, the most relevant change was a significant increase registered in the Fibrobacteres phylum (*P* = 0.019). Accompanied by a (numerical) reduction in Enterobacteriaceae, it could be hypothesized that the supplemented MCFA prompts a more fibrolytic-like adult microbial profile. In addition, it is interesting to remark that several OTUs from the Lachnospiraceae family were decreased with the DIC diet compared to the CTR diet and that the *Lachnospira* genus has been reported to increase with the presence of *Salmonella* [67]. This response of the microbiota to DIC supplementation could also be associated with the response registered in IEL at the ileal level. On d 8 PI, the animals treated with DIC and challenged with *Salmonella* maintained lower numbers of IEL than the CTR group. A possible hypothesis is that the pigs in the DIC group might have retained a more benign environment within the gastrointestinal tract. Another plausible explanation could be that the clear reduction in the pathogen counts in the cecum by the addition of DIC stopped the recruitment of IELs, since these defense cells have been found near *Salmonella* pathogens [68]. In fact, the IEL counts were higher within the *Salmonella* challenge than in previous trials in our group [33, 69], reflecting a more acute course of diarrhea. If we consider that the IELs remain quiescent until facing a real threat [70], it seems reasonable that after the *Salmonella* challenge, DIC treatment had the opportunity to show its effects on the current IEL response.

However, within the ETEC F4 trial, the reduction in possible specific commensal populations such as enterobacteria and coliforms as observed in the ETEC F4-challenged animals, might suggest a more hostile milieu, which consequently mobilized more IELs than the CTR group. In turn, the IEL response could also be related to a possible readaptation of the community structure, as seen in the NMDS results (envfit *P* = 0.035). In this regard, the impact of the MCFA supplementation was evident at the structural level, with the most relevant change in the bacterial groups observed for the increase in *Dialister* (*P* = 0.008) within the Veillonellaceae family. However, other mechanisms related to a direct modulator effect of MCFA on the immune response of the animal should not be disregarded. MCFA supplemented to pre-weaning piglets as triglycerides (MCT) have been shown to stimulate the immune response in the stomach, regardless of the complexity of the microbiota [71]. In addition, similar stimulatory effects on intestinal villi and the local immunity have been demonstrated in LPS-challenged weanlings based on C6- and C8-MCT [23].

To try and elucidate possible associations between the microbial populations and gut variables assessed in this study, particularly those modified by the experimental treatments, we performed a correlation analysis; the most relevant correlations (*r* > 0.5 and adjusted *P* < 0.10) are presented in Table 9. Interestingly, candidate Barnesiellaceae and Enterobacteriaceae families were positively correlated with the IEL counts in the *Salmonella* trial, whereas in the ETEC F4 trial, no significant correlation was found. This could be somehow related to the differential response registered in IEL with DIC between both trials. Valeric acid showed only a significant high inverse correlation with Clostridiaceae (*P* = 0.011) in the *Salmonella* trial. Under the ETEC F4 challenge, valeric acid was positively correlated with *Megasphaera* (*r* = 0.837) and the corresponding family Veillonellaceae (*r* = 0.784). This correlation is coincident with the higher presence of *Dialister* on d 8 PI in DIC animals and the higher percentage of valeric acid than in CTR animals. *Dialister* and *Megasphaera*, among other members within the Veillonellaceae family, have been reported to produce valeric acid as an end metabolite of the fermentation of carbohydrates and lactate [72].

**Table 9:** Pearson’s correlations^a^ between gut variables and bacterial populations for *Salmonella* and ETEC F4 trials

Gut variable	Microbiota^b^	Pearson (*r*)	Adjusted *P*
*Salmonella* trial			
IEL	candidate Barnesiellaceae	0.698	0.055
IEL	candidate Odoribacteraceae	0.694	0.057
IEL	*Odoribacter*	0.694	0.057
IEL	Enterobacteriaceae	0.686	0.065
Valeric acid	Clostridiaceae	-0.782	0.011
ETEC F4 trial			
Valeric acid	*Megasphaera*	0.837	0.005
Valeric acid	Veillonellaceae	0.784	0.023

*IEL* Intraepithelial lymphocytes in the ileal villus

^a^Correlations considered: *r* > 0.5 and adjusted *P* < 0.10

^b^Microbial populations identified by high-throughput sequencing (HTS)

## Conclusions

The results obtained from this study support the activity of a blend of sodium salts of coconut oil distillates in reducing the hindgut colonization by pathogenic populations such as enterobacteria, *E. coli*, and *Salmonella* in orally-challenged weaned piglets. The effects could be mediated by the changes promoted in the microbiota ecosystem since significant effects were registered in different microbial groups. Under a *Salmonella* challenge, significant increases were registered in the Fibrobacteres phylum and after an ETEC F4 challenge, the most relevant changes were registered in the *Dialister* genus of the Veillonellaceae family within a diverging microbial structure. There was also a differential impact of this blend on the intestinal local immune response, leading to higher numbers of ileal intraepithelial lymphocytes after the *Salmonella* challenge, but lower numbers after the ETEC F4 challenge. The effects of the supplemented MCFA might respond to different complex interactions between the opportunistic pathogens, the commensal microbiota and the host response.

## Data Availability

The datasets used and analyzed during the current study are available from the corresponding author on reasonable request. The dataset generated corresponding to the final 16S rRNA gene sequences is available in the European Nucleotide Archive (ENA), accession code PRJEB30494.

## Abbreviations

ADFI: Average daily feed intake
ADG: Average daily gain
ANOSIM: Analysis of similarities
BW: Body weight
CFU: Colony-forming unit
DM: Dry matter
ETEC: Enterotoxigenic *Escherichia coli*
FM: Fresh matter
GC: Goblet cells
HTS: High-throughput sequencing
IEL: Intraepithelial lymphocytes
MCFA: Medium-chain fatty acids
MCT: Medium-chain triglycerides
MIT: Mitoses
OTU: Operational taxonomic unit
PBS: Phosphate buffered solution
PI: Post-inoculation
PreI: Pre-inoculation
qPCR: Quantitative polymerase-chain reaction
SCFA: Short-chain fatty acids
